# Connectivity of the insular subdivisions differentiates posttraumatic headache-associated from nonheadache-associated mild traumatic brain injury: an arterial spin labelling study

**DOI:** 10.1186/s10194-024-01809-z

**Published:** 2024-06-19

**Authors:** Fengfang Li, Di Zhang, Jun Ren, Chunhua Xing, Lanyue Hu, Zhengfei Miao, Liyan Lu, Xinying Wu

**Affiliations:** Department of Radiology, Nanjing First Hospital, Nanjing Medical University, No.68, Changle Road, Nanjing, 210006 China

**Keywords:** Posttraumatic headache, Mild traumatic brain injury, Insular subdivisions, Functional connectivity, Arterial spin labelling

## Abstract

**Objective:**

The insula is an important part of the posttraumatic headache (PTH) attributed to mild traumatic brain injury (mTBI) neuropathological activity pattern. It is composed of functionally different subdivisions and each of which plays different role in PTH neuropathology.

**Methods:**

Ninety-four mTBI patients were included in this study. Based on perfusion imaging data obtained from arterial spin labelling (ASL) perfusion magnetic resonance imaging (MRI), this study evaluated the insular subregion perfusion-based functional connectivity (FC) and its correlation with clinical characteristic parameters in patients with PTH after mTBI and non-headache mTBI patients.

**Results:**

The insular subregions of mTBI + PTH (mTBI patients with PTH) and mTBI-PTH (mTBI patients without PTH) group had positive perfusion-based functional connections with other insular nuclei and adjacent discrete cortical regions. Compared with mTBI-PTH group, significantly increased resting-state perfusion-based FC between the anterior insula (AI) and middle cingulate cortex (MCC)/Rolandic operculum (ROL), between posterior insula (PI) and supplementary motor area (SMA), and decreased perfusion-based FC between PI and thalamus were found in mTBI + PTH group. Changes in the perfusion-based FC of the left posterior insula/dorsal anterior insula with the thalamus/MCC were significant correlated with headache characteristics.

**Conclusions:**

Our findings provide new ASL-based evidence for changes in the perfusion-based FC of the insular subregion in PTH patients attributed to mTBI and the association with headache features, revealing the possibility of potential neuroplasticity after PTH. These findings may contribute to early diagnosis of the disease and follow-up of disease progression.

## Introduction

Posttraumatic headache (PTH) is a secondary headache disorder defined by the third version of the International Classification of Headache Disorders (ICHD-3) as a headache caused by underlying head and/or neck trauma or injury [1, 2]. This condition, which can cause severe pain and disability, is one of the most common symptoms after mild traumatic brain injury (mTBI) and one of the most likely symptoms to persist after mTBI [3, 4]. Previous studies have reported that the cumulative incidence of PTH within 1 year after mTBI is as high as 91% [5, 6]. Although PTH may be the only symptom after a head injury, it is often accompanied by other trauma-related symptoms, including sleep problems, posttraumatic stress, imbalance or vertigo, cognitive dysfunction, personality changes, depression, anxiety, and extracranial pain [7–9]. However, no specific symptoms or headache features have been shown to contribute to the diagnosis of PTH [10]. The high prevalence and impact of PTH emphasize the need for further exploration of its pathophysiological mechanism, its early recognition and accurate diagnosis, and early effective intervention for and prognostic evaluation of patients with PTH. However, the underlying pathophysiological mechanisms of PTH remain unclear, and there are currently no evidence-based methods for specifically diagnosing and evaluating PTH.

Because of its extensive structural connections with the anterior cingulate cortex, olfactory cortex, orbitofrontal cortex, amygdala, and superior temporal sulcus, the insula has traditionally been described as the paralimbic cortex or “limbic integration cortex” [11]. Functional neuroimaging studies have shown that the anterior and posterior functions of the insula are spatially distinct. For example, the anterior insular cortex (AIC) is active when tracking and sensing pain, while the posterior insular cortex (PIC) is involved in harmless temperature perception [12, 13]. More recently, the “pain matrix” has been redefined as the cortical network that responds to nociceptive stimuli and includes the somatosensory cortex, the insular cortex, and the medial cingulate cortex. Pain—whether chronic or acute, physical or psychological—activates the AIC [14]. PTH can cause damage to the insula, a finding of special interest to researchers in recent years. Nikolova et al. showed that the T2* value of the left insular cortex in PTH patients was lower than that in healthy controls, and multiple linear regression showed that the T2* value of the insula was negatively correlated with the frequency of headache [15]. A functional MRI (fMRI) studyrevealed significant differences in static functional connectivity (FC) in the PIC between patients with PTH and patients with migraine. The findings also showed that headache frequency in the PTH group was correlated with static FC in the right posterior insula and the left cingulate [16]. However, to the best of our knowledge, insular subregional FC in patients with MTBI-induced PTH has not been studied to date.

In addition, the abovementioned studies were mainly based on fMRI signals obtained from imaging sequences sensitive to blood oxygen level-dependent (BOLD) contrast. Recently, however, studies have evaluated perfusion-based FC based on data collected from perfusion MRI via resting-state arterial spin labelling (ASL) [17]. Due to the tight coupling of neural activity with regional perfusion, changes in underlying brain activity can be reflected by fluctuations in the perfusion signals. At resting state, the perfusion power spectrum is essentially flat, while the BOLD power spectrum shows increased noise power at low frequencies [18]. Therefore, ASL may better characterize low-frequency fluctuations than BOLD signals [19, 20]. One study showed that ASL demonstrats comparable repeatability and statistical power to BOLD fMRI [21].

In this study, based on previous evidence, we used MRI based on resting-state ASL and seed-based methods to investigate global brain perfusion-based FC changes in the insular subregion in PTH and nonheadache mTBI patients and then explored the relationship between insular subregion perfusion-based FC abnormalities and the clinical characteristics of PTH. We hypothesize that the perfusion-based FC of the insular subdivision with other cortical areas in PTH patients would be altered with respect to that in nonheadache mTBI patients. Finally, receiver operating characteristic (ROC) curve analysis was used to evaluate the ability of insular subregion perfusion-based FC measurements to distinguish PTH from nonheadache mTBI patients.

## Methods

### Subjects

Right-handed mTBI patients were prospectively enrolled in this study. mTBI was defined according to the guidelines of the American Congress of Rehabilitation Medicine [22]. The inclusion criteria were age 18 years or older, Glasgow Coma Scale (GCS) score of 13–15, posttraumatic amnesia lasting < 24 h, and loss of consciousness < 30 min. The exclusion criteria were as follows: (a) previous history of head injury or psychiatric/neurological disease; (b) history of drug, alcohol or substance abuse; (c) contraindications to MRI; and (c) history of migraine or any other headache prior to injury. All mTBI patients initially underwent MRI scans and clinical and neurological scale assessments within 7 days after injury (3.06 ± 2.03 days). According to the diagnostic criteria of the ICHD-3 beta [23], mTBI patients were divided into two groups: mTBI with and without PTH. All patients underwent clinical and neuropsychological follow-up at 3 months after injury. All patients with PTH were assessed for the sites of major headache, headache intensity, and headache frequency (days/months). Headache intensity was assessed using a visual analogue scale (VAS) [24], a numerical rating scale from 0 to 10, where 0 represented no pain and 10 represented the most severe pain imaginable. Neurocognitive status was assessed using the Montreal Cognitive Assessment Method (MoCA) [25]. The MoCA, a sensitive cognitive screening test often administered after mTBI, has been widely used in patients with pain disorders, according to Santangelo et al. [26]. The maximum score on the test is 30; a score greater than 26 is considered normal, while lower scores indicate greater cognitive deficits. This study was approved by the Institutional Review Board of Nanjing Medical University, and all participants provided written informed consent.

### MRI data acquisition

Data acquisition was carried out on a 3.0 T MRI scanner (Ingenia, Philips Medical Systems, Netherlands) using an 8-channel standard head coil. ASL data were collected using a 2D-pseudocontinuous ASL (pCASL) sequence: repetition time (TR) = 4000 ms; echo time (TE) = 11 ms; postlabel delay = 1600 ms; label duration = 1650 ms; flip angle (FA) = 90°; slice thickness = 5 mm with 10% gap; field of view (FOV) = 240 mm × 240 mm; matrix = 64 × 64; 30 control/label pairs; the Z-direction was foot-head, and an ASL scan duration of 4 min and 8 s. Anatomical data were collected using a three-dimensional turbo fast-echo (3D-TFE) T1-weighted imaging (T1WI) sequence: TE/TR = 3.7 ms/8.1 ms; slices = 170; thickness = 1 mm; FA = 8°; gap = 0 mm; FOV = 256 mm×256 mm; acquisition matrix = 256 × 256; and a total scan duration of 5 min and 28 s. Susceptibility weighted imaging (SWI) data were obtained using a 3D gradient echo (GRE) sequence: TE/TR = 34 ms/22 ms; FA = 20; slice thickness = 1 mm; FOV = 220 mm×220 mm; and matrix = 276 × 319.

### MRI data preprocessing

Data preprocessing was carried out using Statistical Parametric Mapping (SPM8, http://www.fil.ion.ucl.ac.uk/spm) and the ASL data processing toolbox ASLtbx (https://cfn.upenn.edu/~zewan). The labelled and control images were realigned, coregistered with the anatomical data set, and subtracted in pairs to obtain a time series of perfusion images. The detailed procedures have been described in a previous study [27]. In short, first, motion correction was carried out, and data with motion translations > 2 mm or rotations > 2° were removed. Second, the obtained data were registered to the structural images. Then, the data were normalized to the Montreal Neurological Institute (MNI) standard space (resolution: 2 × 2 × 2 mm^3^). Finally, spatial smoothing was performed with a 6-mm full-width at half-maximum (FWHM) Gaussian kernel.

### Perfusion-based FC analysis

**Fig. 1:**
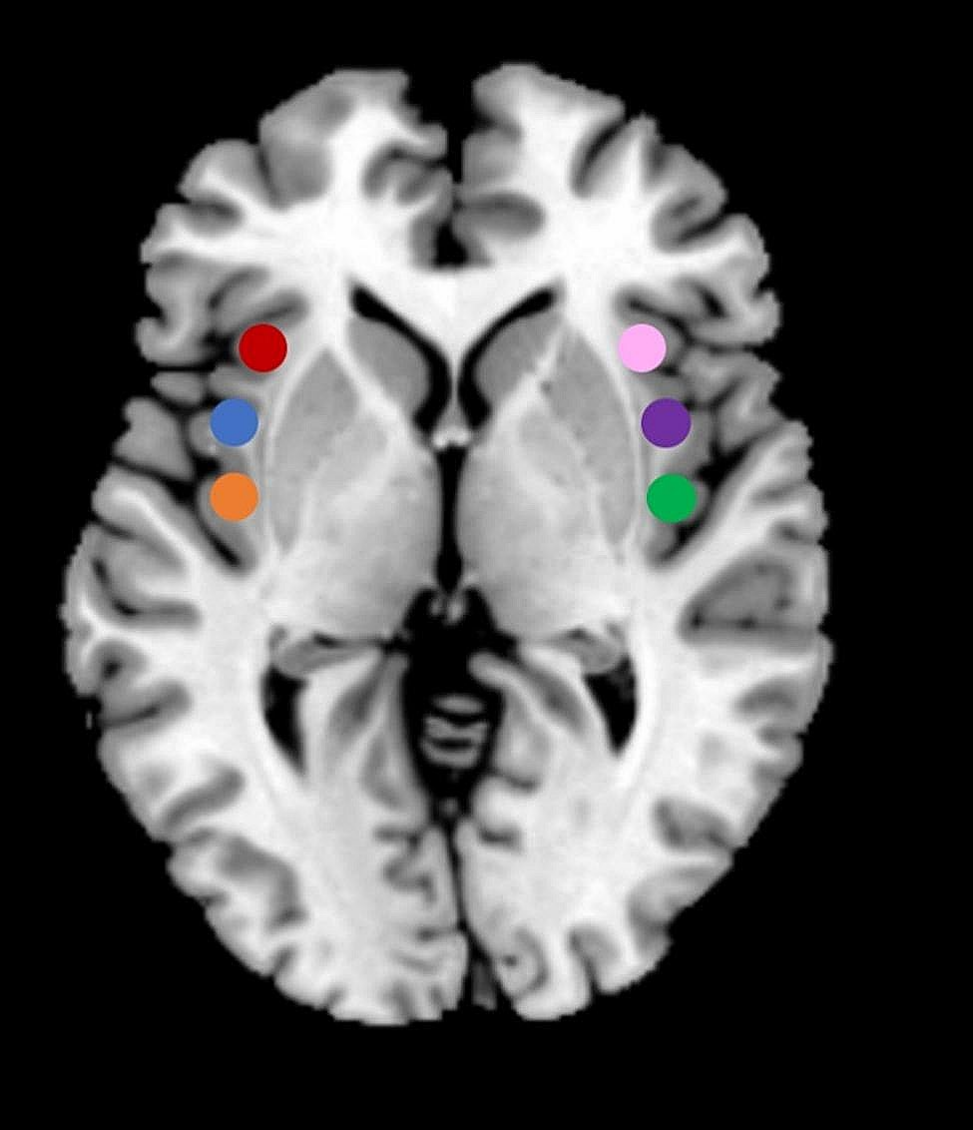
Subdivisions of the insular cortex: right ventral anterior insula (R-vAI, red), right dorsal anterior insula (R-dAI, blue), right posterior insula (R-PI, orange), left ventral anterior insula (L-vAI, pink), left dorsal anterior insula (L-dAI, purple), and left posterior insula (L-PI, green)

Perfusion-based FC analysis was performed using the Data Processing & Analysis of Brain Imaging (DPABI, http://rfmri.org/DPABI) toolbox to assess the temporal correlations of slow spontaneous fluctuations in the resting cerebral blood flow (CBF) time series. Six spherical seeds (radius = 6 mm) were centred on the coordinates of each region of interest (ROI) in the MNI 152 space (Fig. 1). Based on previous study [28], the identities and coordinates of the resulting six ROIs are as follows: right ventral anterior insula (R-vAI, red, MNI = 32, 10, − 6), right dorsal anterior insula (R-dAI, blue, MNI = 35, 7, 3), right posterior insula (R-PI, orange, MNI = 35, − 11, 6), left ventral anterior insula (L-vAI, pink, MNI = − 33, 13, − 7), left dorsal anterior insula (L-dAI, purple, MNI = -38, 6, 2), and left posterior insula (L-PI, green, MNI = − 38, − 6, 5). A similar seed-based method was used for perfusion-based FC analysis by selecting six ROIs located in the insular subdivision as seeds. Prior to Pearson correlation analysis, several covariates (translation (x, y, z) and rotation (α, β, γ) motion parameters) were removed from the data by linear regression to eliminate spatial correlations due to noise. Then, the signal in the CBF time series was low-pass filtered (f < 0.07 Hz) [19]. Since the CBF time series is not affected by the low-frequency drift present in the BOLD data, a high-pass filter is not used. Pearson’s correlation was performed to estimate the voxelwise seed-to-voxel connectivity of each subject, and Fisher’s r-to-z transformation was subsequently performed. Six individual Z-maps were obtained, representing the strength of the correlation between each seed and each brain voxel. These maps were then used for a second level of random effects analysis.

### Statistical analyses

The Kolmogorov–Smirnov test was carried out to assess the normality of the distribution of the demographic data. Demographic characteristics and clinical data were compared between groups using two-sample t tests (mean values) and χ-square tests (sex ratios). *P* < 0.05 was taken as the threshold for statistical significance. The group-level one sample T test was used to evaluate the perfusion-based FC maps of the 6 insula subdivision ROIs in each group, and the group connectivity maps of the insula subnetwork in each group were obtained based on the CBF (false discovery rate (FDR) correction, *p* < 0.05). For each ROI, a 2-sample t test was conducted to compare the perfusion-based FC maps of the insular subdivisions between mTBI patients with PTH and mTBI patients without PTH while controlling for age, sex, and educational level (Gaussian random field correction, clustering level *p* < 0.05, voxel level *p* < 0.001). Spearman’s correlation analysis was used to evaluate the relationships between significant cluster perfusion-based FC values and clinical variables, controlling for age, sex, and educational level (Bonferroni correction, *p* < 0.05). ROC curve analysis and binary logistic regression were used to assess the extent to which perfusion-based FC z values extracted from clusters with significant differences between groups were able to distinguish PTH patients from non-PTH patients. The differentiation performance of each cluster perfusion-based FC index was evaluated. In addition, a binary logistic regression model was constructed by combining all the indexes, and its performance was evaluated.

## Results

### Demographics

A total of 94 patients with mTBI were included in this study. The participants were divided into two groups: the mTBI + PTH group (mTBI patients with PTH, *n* = 48) and the mTBI-PTH group (mTBI patients without PTH, *n* = 46). The two groups were well matched for age (*p* = 0.191), sex (*p* = 0.409), and educational level (*p* = 0.206). There was no significant difference in the MoCA score (*p* = 0.364) between the groups. The demographic information of the participants is shown in Table 1. No visible traumatic lesions were observed on SWI.

**Table 1:** Demographic and cognitive variables of the mTBI with PTH patients and mTBI without PTH patients

Characteristics	mTBI + PTH (*n* = 48)		mTBI-PTH (*n* = 46)	*p*-value
Age (years)		39.00 ± 11.05	42.13 ± 11.98	0.191
Education (years)		13.10 ± 3.06	14.02 ± 3.88	0.206
Gender (Female/ Male)		21/27	25/21	0.409
MoCA scores		24.46 ± 2.72	24.98 ± 2.80	0.364
Headache characteristics				
Predominant side				
Right		18	-	-
Left		12	-	-
Bilateral		18	-	-
Unilateral		30	-	-
Headache frequency		11.69 ± 7.25	-	-
Headache intensity		4.62 ± 1.90	-	-

The data are shown as the mean ± SD. mTBI, mild traumatic brain injury; PTH, posttraumatic headache; GCS Glasgow Coma Scale; MoCA, Montreal Cognitive Assessment

### Perfusion-based insular FC maps in mTBI + PTH patients and mTBI-PTH patients

**Fig. 2:**
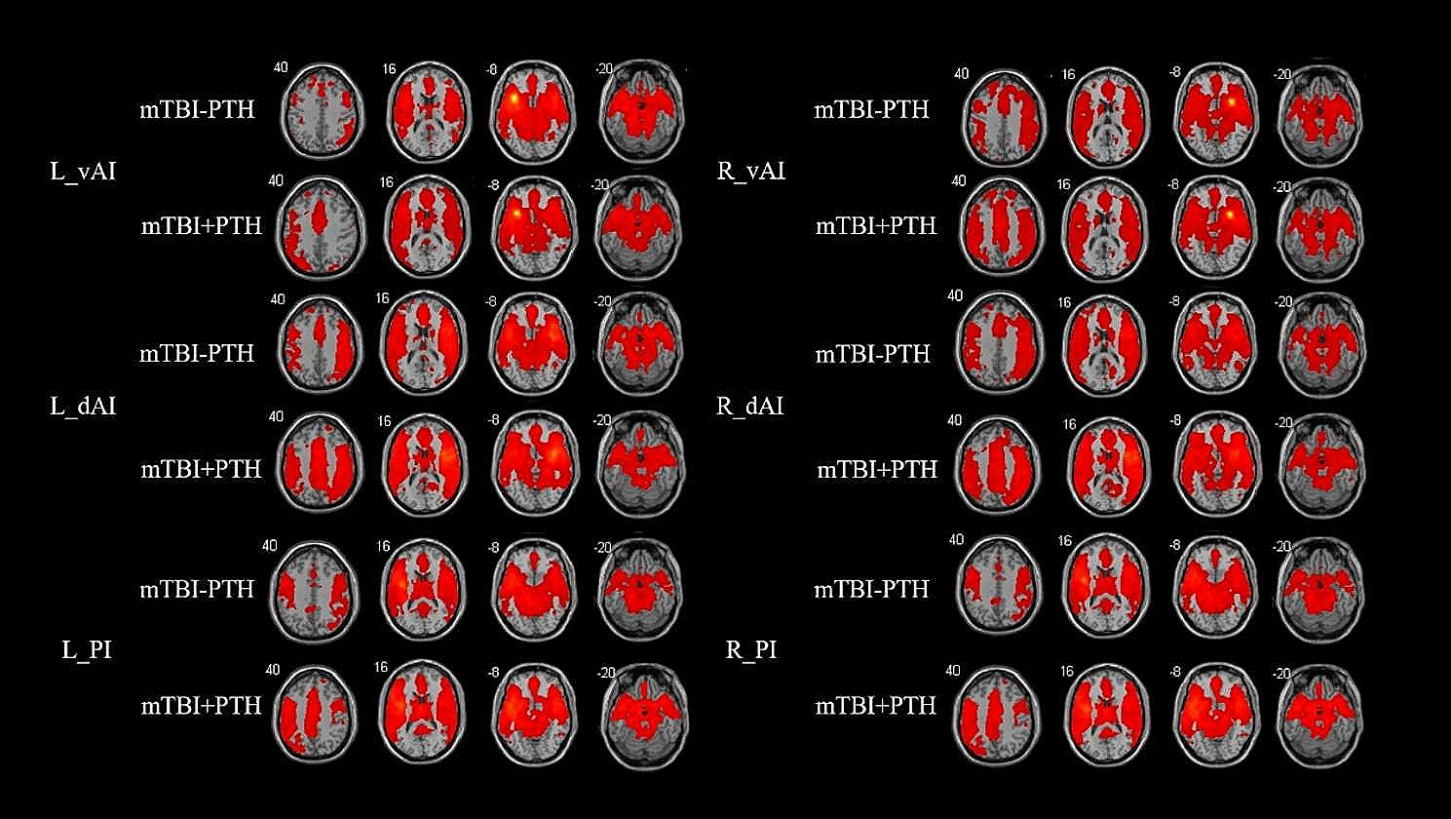
Perfusion-based functional connectivity maps of six insular subdivisions (R-vAI, R-dAI, R-PI, L-vAI, L-dAI, L-PI) in mTBI + PTH patients (mTBI patients with PTH, *n* = 48) and mTB-PTH patients (mTBI patients without PTH, *n* = 46). L, left; R, right; vAI, ventral anterior insula; dAI, dorsal anterior insula; PI, posterior insula

The perfusion-based FC distributions of the insular subregions were largely similar in the right and left hemispheres in both mTBI + PTH patients and mTBI-PTH patients (Fig. 2). Functionally, all the insular subdivisions had positive perfusion-based FC with most of the other insular and adjacent brain regions, including the frontal, parietal, and temporal lobes. No significant negative perfusion-based FC was found between the insular subregions and other brain regions in any of the groups.

### Perfusion-based FC of mTBI + PTH patients versus mTBI-PTH patients

**Fig. 3:**
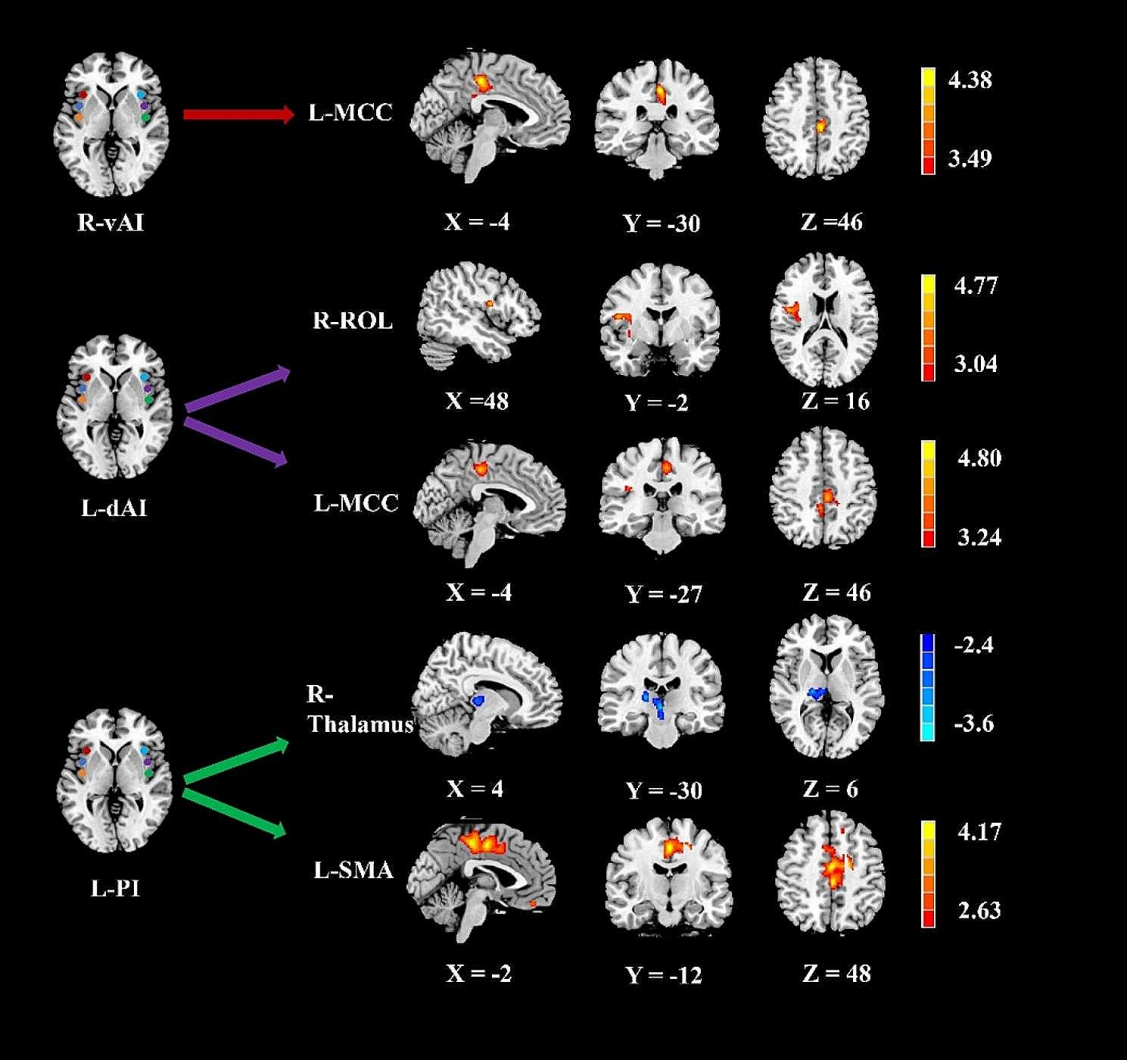
Group differences in perfusion-based functional connectivity for 3 ROI (R-vAI, L-dAI, L-PI) (Gaussian random field correction, clustering level *p* < 0.05, voxel level *p* < 0.001). The cold color indicates decreased CBF and hot color indicates increased CBF in mTBI + PTH patients compared to mTB-PTH patients. L, left; R, right; vAI, ventral anterior insula; dAI, dorsal anterior insula; PI, posterior insula; MCC, middle cingulate cortex; SMA, supplementary motor area; ROL, Rolandic operculum

Figure 3; Table 2 show the significant differences in perfusion-based FC between mTBI + PTH and mTBI-PTH patients. mTBI + PTH patients showed changes in perfusion-based FC associated with the insular subregion, mainly in the connectivity with the cingular-opercular network and sensorimotor network. The perfusion-based FC of the insular subregions increased in four clusters in the mTBI + PTH group; specifically, compared with that in the mTBI-PTH group, the perfusion-based FC between the left dAI and the right Rolandic operculum (ROL)/left middle cingulate cortex (MCC) was significantly greater in the mTBI + PTH group, as was the perfusion-based FC between the right vAI and the left MCC and between the left PI and the left supplementary motor area (SMA). Additionally, the perfusion-based FC between the left PI and the right thalamus was significantly lower in the mTBI + PTH group. No significant changes in the perfusion-based FC of the other insular subdivisions (L_vAI, R_PI, R_dAI) were found between two groups.

**Table 2:** Brain regions with significant group differences in the perfusion-based functional connectivity of insular

Brain regions	BA	Peak MNI coordinates x, y, z (mm)	Peak T value	Cluster size(Voxels)	
**ROI: R_vAI**					
L_MCC	31	-4, -30, 46	4.5609	291	
**ROI: L_PI**					
L_SMA	24	-2, -12, 48	4.4693	620	
R_Thalamus		4, -30, 6	-3.8932	517	
**ROI: L_dAI**					
R_ROL	13	48, -2, 16	4.5567	360	
L_MCC	31	-4, -27, 46	4.4424	410	

MNI, Montreal Neurological Institute. R_vAI, right ventral anterior insula; L_PI, left posterior insula; L_dAI, left dorsal anterior insula; MCC, middle cingulate cortex; SMA, supplementary motor area; ROL, Rolandic operculum; L, left; R, right

### Correlations of insula subdivision perfusion-based FC with clinical variables

The perfusion-based FC between the left dAI and left MCC was negatively correlated with headache intensity (*r* =-0.388, *p* = 0.006), while the perfusion-based FC between the left PI and right thalamus was positively associated with headache frequency (*r* = 0.357, *p* = 0.012).

### Discriminatory performance of the perfusion-based FC indexes

**Fig. 4:**
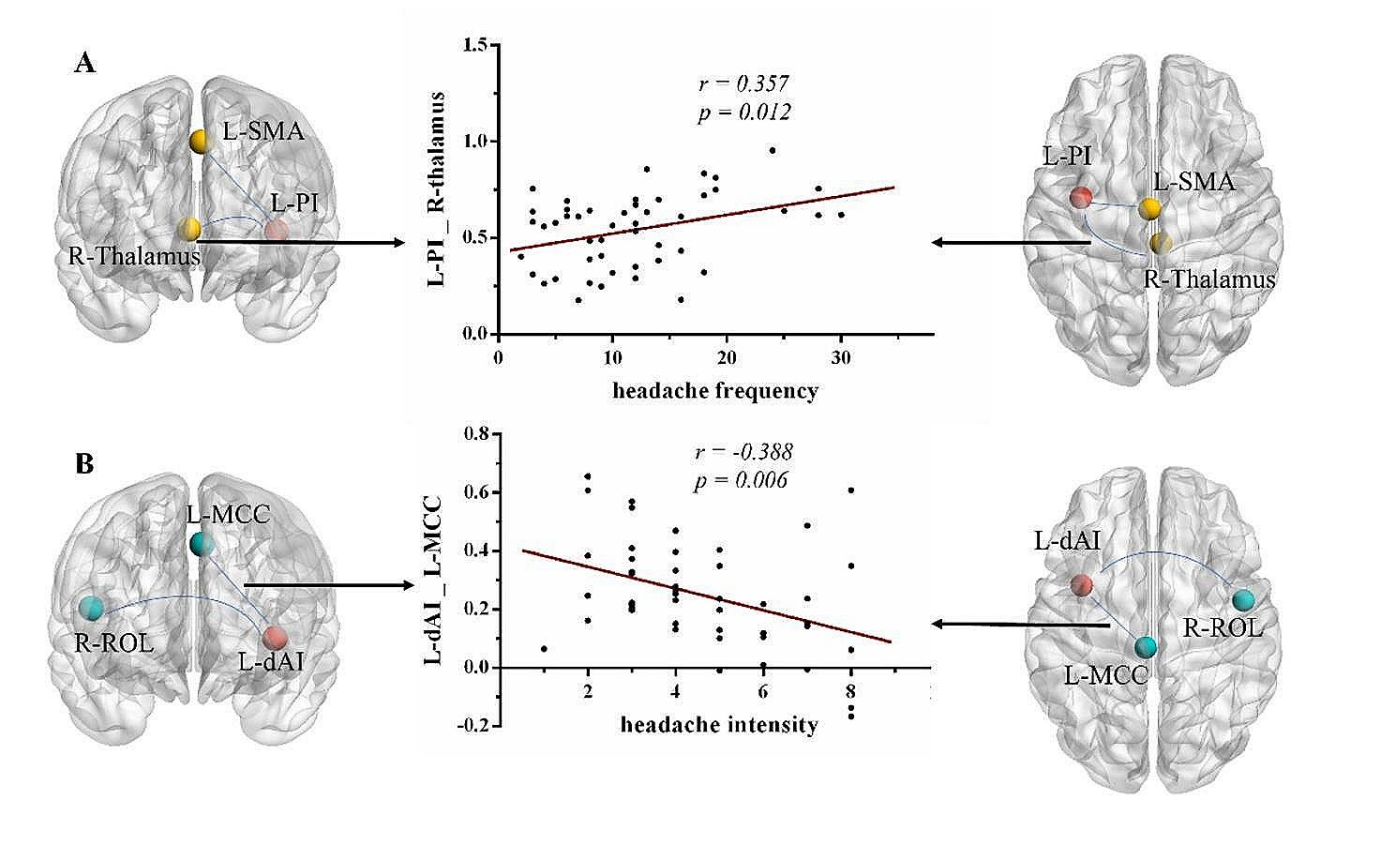
Correlations between perfusion-based functional connectivity changes and headache characteristics in mTBI + PTH group. (A) Correlations between FC changes (between the L-PI and the R-thalamus) and headache frequency. (B) significant correlation between FC changes (between the L-dAI and the L-MCC) and headache intensity score. L, left; R, right; dAI, dorsal anterior insula; PI, posterior insula; MCC, middle cingulate cortex

**Fig. 5:**
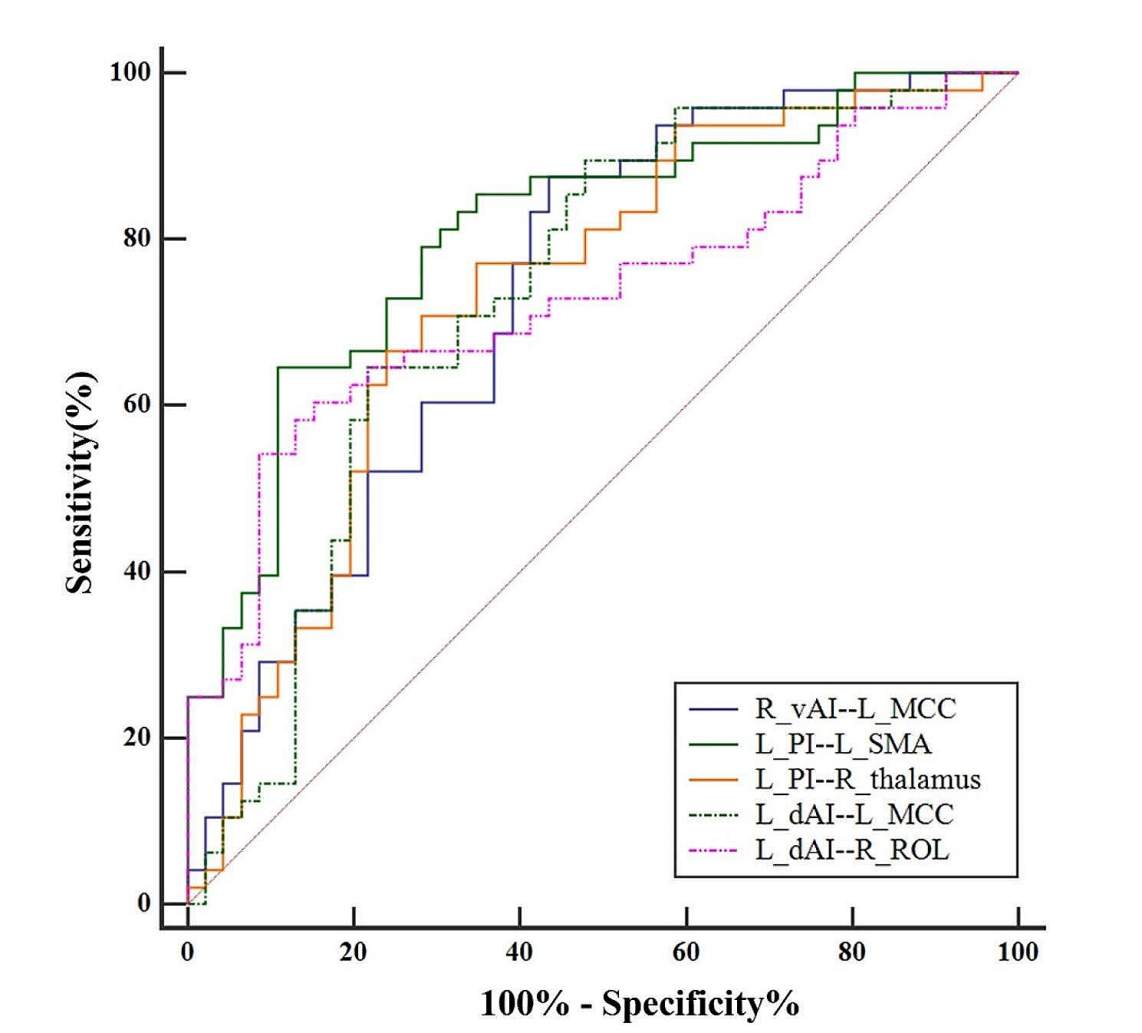
ROC curves of the significant functional connectivity of ROIs for mTBI + PTH group and mTBI-PTH group. ROC, receiver operating characteristic. R_vAI, right ventral anterior insula; L_PI, left posterior insula; L_dAI, left dorsal anterior insula; MCC, middle cingulate cortex; SMA, supplementary motor area; ROL, Rolandic operculum

ROC curve analysis revealed that the perfusion-based FC between the left PI and the left SMA best discriminated between the mTBI + PTH and mTBI-PTH groups, with an area under the curve (AUC) of 0.809 (95% CI = 0.715–0.883; Figs. 4 and 5). Additionally, the binary logistic regression model constructed from five connectivity indicators had the greatest ability to distinguish mTBI + PTH and mTBI-PTH patients, with an AUC of 0.882 (95% CI = 0.799–0.939; Table 3).

**Table 3:** Results of ROC for the discrimination between mTBI + PTH and mTBI-PTH patients

Functional connectivity index	AUC (95%CI)
Right vAI – left MCC	0.732(0.631–0.818)
Left PI – left SMA	0.809(0.715–0.883)
Left PI – right thalamus	0.733(0.632–0.819)
Left dAI – left MCC	0.735(0.634–0.820)
Left dAI – right ROL	0.731(0.630–0.818)
Combination of five indexes	0.882(0.799–0.939)

AUC, the area under the ROC curve; CI, confidence intervals; R_vAI, right ventral anterior insula; L_PI, left posterior insula; L_dAI, left dorsal anterior insula; MCC, middle cingulate gyrus; SMA, supplementary motor area; ROL, Rolandic operculum

## Discussion

In this study, the resting-state perfusion-based FC changes in the insular subregion were investigated both in patients with PTH attributed to mTBI and in patients with mTBI without PTH using fMRI techniques based on ASL perfusion. This study confirmed that both increases and decreases in resting-state perfusion-based FC occur in the insular subregion of patients with PTH caused by mTBI, revealing changes in the perfusion-based FC of the left dAI, left PI, and right vAI after PTH, mainly with the cingular-ocular network (CON) and sensorimotor network (SMN), extending beyond the traditional pain processing area. Additionally, there were significant correlations between headache features and perfusion-based FC changes between the left dAI and MCC and between the left PI and right thalamus.

Emerging evidence shows that the insula, as a key hub of the SN, is an interoceptive signal receiver that plays a central role in the coordination of neural resources, dynamic cognitive control, and detection of behavioural stimuli [29]. Subdivisions of the insula based on whole-brain perfusion-based FC patterns showed that the anterior insula (AI) is mainly connected to the dorsal anterior cingulate, while the PI is mainly connected to the SMA and middle cingulate, which has also been reflected in anatomical connectivity patterns [30, 31]. The AI tracks emotions (e.g., disgust) and perceptions (e.g., pain and temperature) associated with physical states, and the AIC is active when it perceives pain and heat intensity [32]. Consistent with previous evidence, we found significant increases in perfusion-based FC between the dAI and MCC and between the vAI and MCC in the PTH group relative to the mTBI-PTH group. Similarly, significant differences in static FC between patients with PTH and patients with migraine were also found for several region pairs, including the insula and MCC, based on BOLD resting-state imaging data [16]. In addition, we observed that perfusion-based FC changes between the left dAI and left MCC were negatively correlated with headache intensity in patients with PTH, which highlights the important role of the dAI. Dumkrieger et al. ‘s study based on bold MRI also showed that FC between the right posterior insula and the left hypothalamus was significantly negatively correlated with headache frequency in patients with PTH [16]. Moreover, for PTH patients, the increase in perfusion-based FC between the dAI and MCC may represent abnormal activity of the “pain matrix”, composed of the AIC, cingulate cortex, and somatosensory cortex and may be one of the neuropathological bases for PTH formation. However, whether these perfusion-based FC changes are inherent in the brain after PTH is unknown and needs to be further explored in future studies.

Specifically, the PTH group showed increased perfusion-based FC between the dAI and Rol in this study. The ROL, as an essential brain region of the CON, is mainly involved in task initiation, monitoring and maintenance processes and has a role in complex functions, mainly movement, autonomy, sensation, and emotional and cognitive processing [33, 34]. A meta-analysis of voxel-based morphometric (VBM) studies reported that migraine patients showed grey matter abnormalities in the left insula and the bilateral ROL [33] [35]. Another study based on resting-state BOLD fMRI showed that patients with acute mTBI had less-effective connections from the left insula to the right Rol than healthy controls [36]. Previous studies have shown that there are close structural and functional connections between the insula and the Rol [37]. Moreover, Dong L et al. reported that chronic migraine patients showed changed mean amplitude of low-frequency fluctuation in the right ROL [38]. Therefore, we speculate that perfusion-based FC changes between the dAI and Rol may play an important role in the occurrence and development of PTH after mTBI. However, the specific role of ROL in PTH, whether it is related to pain or accompanying cognitive impairment, is unclear.

The PI is a granular region that receives input from the occipital, parietal, thalamic, and temporal cortices and is closely associated with sensorimotor and pain processes. The PI mainly receives and integrates various interoceptive signals and then sends the signals to the AI [39]. The PI network components identified by FC analysis include the auxiliary motor area and somatosensory cortex [40]. Consistent with previous findings, we found a significant increase in perfusion-based FC between the PI and left SMA in patients with PTH, which is also partially consistent with the findings of Li et al., who observed increased effective connectivity of the left insula, primarily with the left motor cortex, in acute mTBI patients [36]. Another study by Nikolova and colleagues revealed that individuals with PTH exhibited abnormal magnetic resonance T2* values compared with healthy controls in the right SMA, right Rol, and left insula [15]. The second cortical area whose perfusion-based FC with the PI was altered in PTH patients was the right thalamus, and the strength of this perfusion-based FC was found to be correlated with headache frequency in the PTH group. A study based on multimodal T1-weighted and T2* brain MRI showed lower T2* values in the left thalamic subfield (indicating more iron deposition) in PTH patients than in healthy controls and a significant negative correlation between T2* signals in the left thalamus and headache intensity [41]. Previous studies based on the pcasl approach examined changes in CBF in chronic migraine patients, showing abnormal CBF and arterial cerebral blood volume in the bilateral ventral nucleus region of the thalamus compared to healthy subjects [42, 43]. These findings are consistent with data from the literature supporting an important role of the thalamus in headache attack [44]. The thalamus plays a key role in the perceptual integration and the pathological mechanism of headache, but its role in PTH is unclear [45]. This result highlights the important role of the thalamus, and we speculate that perfusion-based FC alterations between the PI and thalamus may be one of the foundations of PTH. Future studies should be conducted to confirm these abnormalities and possibly provide additional evidence for other imaging techniques to report changes in the PI-thalamic FC in PTH patients, thus indicating whether this region is specifically affected in PTH.

Despite directly reflecting regional neuronal activity, there is little research on whether perfusion-based FC calculated based on ASL data can potentially identify or predict patients with PTH caused by mTBI. In the current study, we found that the perfusion-based FC strength between the left PI and left SMA was able to distinguish between mTBI + PTH and mTBI-PTH patients well. Our results provide preliminary evidence that the perfusion-based FC in the insular subregion may be useful as a perfusion biomarker for assessing PTH. Although they have not yet been validated in separate groups, we believe that indicators of insular subregion perfusion-based FC derived from ASL data may aid in the diagnosis of early PTH and thus deserve further study.

This study has several limitations. First, the sample size of this study was relatively small, which may have affected the statistical power. Future follow-up studies with larger data sets are needed to validate the findings of this paper. Second, this study lacked a matched healthy control group that could be directly compared with the mTBI + PTH/mTBI-PTH groups. Further studies are needed to compare the structural and functional FC of insular subregions between mTBI + PTH and mTBI-PTH groups and matched healthy controls. Third, we used an ROI approach based on a priori defined regions in this study and did not evaluate large-scale network effect changes. Future studies are needed to investigate the potential changes in perfusion-based FC in the early stages of PTH, both horizontally and longitudinally, and their relationship to various clinical features in patients with PTH. Fourth, this study did not analyze static (mean) perfusion coupling differences and mean CBF differences between the two groups. It is necessary to conduct this analysis in future studies. Finally, it is difficult to confirm whether the PTH-related perfusion-based FC changes identified in the insula subregion are risk factors for disease or subsequent acquired changes in the current study and other cross-sectional analyses. More longitudinal studies are needed to further explore this question in the future.

## Conclusions

Based on ASL data, this study revealed abnormal changes in insular subregion-cortical perfusion-based FC in specific brain areas of PTH patients after mTBI as well as correlations between abnormal perfusion-based FC and headache characteristics. We have provided new evidence for perfusion-based FC changes in the insula subregion in PTH patients after mTBI, revealing potential neural remodeling and adaptation of the insula central circuit after PTH, as well as the possibility of a potential early predictive biomarker that could aid in early diagnosis of the disease and follow-up of disease progression.

## Data Availability

No datasets were generated or analysed during the current study.

## Abbreviations

fMRI: Functional magnetic resonance imaging
PTH: Posttraumatic headache
mTBI: Mild traumatic brain injury
ASL: Arterial spin labeling
FC: Functional connectivity
vAI: Ventral anterior insula
dAI: Dorsal anterior insula
PI: Posterior insula
MCC: Middle cingulate cortex
SMA: Supplementary motor area
ROL: Rolandic operculum
BOLD: Blood oxygen level correlation
ROC: Receiver operating characteristic
GCS: Glasgow Coma Score
MoCA: Montreal Cognitive Assessment
VAS: Visual analogue scale
SMN: Sensorimotor network
CON: Cingular-ocular network
